# Supplementary material for: SERPINE1 rs6092 Variant Is Related to Plasma Coagulation Proteins in Patients with Severe COVID-19 from a Tertiary Care Hospital
Source: Biology (Basel). 2022 Apr 14;11(4):595. doi: 10.3390/biology11040595 (PMC9029072; doi:10.3390/biology11040595)
Supplement: Supplementary file 1 [file biology-11-00595-s001.zip › biology-1631819-supplementary.pdf]

Supplementary Material

Table S1. Logistic regression analysis of *SERPINE1* rs6092 including co-variables in IMV *vs* Non-IMV groups.

TEST	*p*-value	OR	95% CI
rs6092 additive effect model	0.9994	1.40E+04	0
Covar 1: Leukocytes	8.82E-09	1.235	1.149-1.327
Covar 2: Platelets	0.0104	0.998	0.996-0.999
2df test of the SNV whilst controlling for covariables	0.7864	NA	NA

IMV: invasive mechanical ventilation; NA: does not apply; SNV: Single nucleotide variant; OR: Odds ratio; 95% CI: 95% Confidence interval.

Table S2. Evaluation of differences in coagulation proteins' levels according to the gender of patients

Protein (pg/mL)	Female (n=51)	Male (n=80)	*p*-value*
P-Selectin	510 [253-843]	418 [255-1,037]	0.8006
D-dimer	534 [335-860]	471 [263-1,135]	0.9135
PSGL-1	5,944 [4,559-7,122]	5,944 [4,999-7,034]	0.5807
tPA	2,000 [1,285-3,178]	2,186 [1,469-3,423]	0.5872
PAI-1	77,879 [73,811-85,457]	80,600 [73,166-87,347]	0.7860
Factor IX	17,635 [13,863-23,148]	16,961 [14,287-20,742]	0.3660

Proteins' levels are presented as median [interquartile range]. *Mann-Whitney U Test. Statistically significant values are presented in bold style. PAI-1, plasminogen activator inhibitor-1; PSGL-1, P-selectin glycoprotein ligand-1; tPA, tissue plasminogen activator.

Supplementary Figures

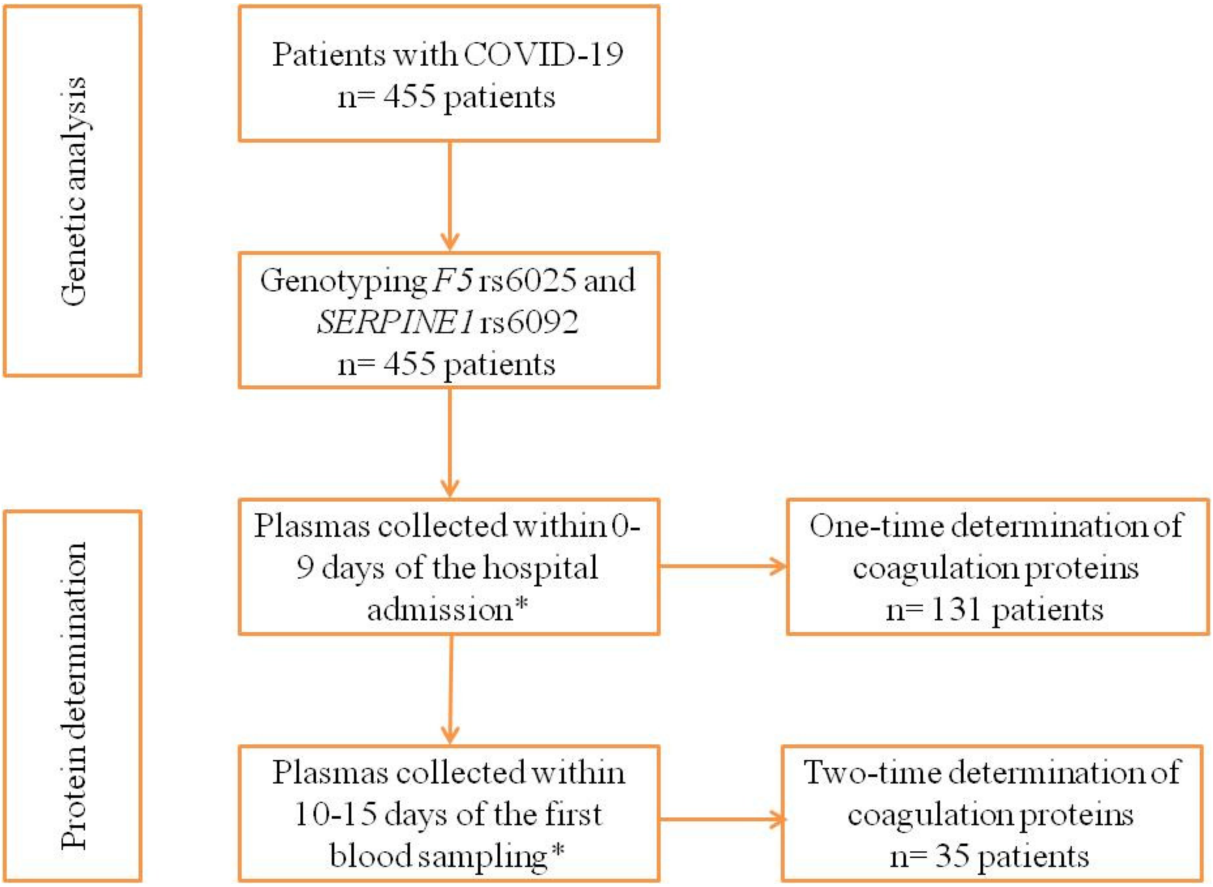

Figure S1. Selection of patients for the coagulation proteins determination.
*The genotype of *F5* rs6025 and *SERPINE1* rs6092 was also considered.

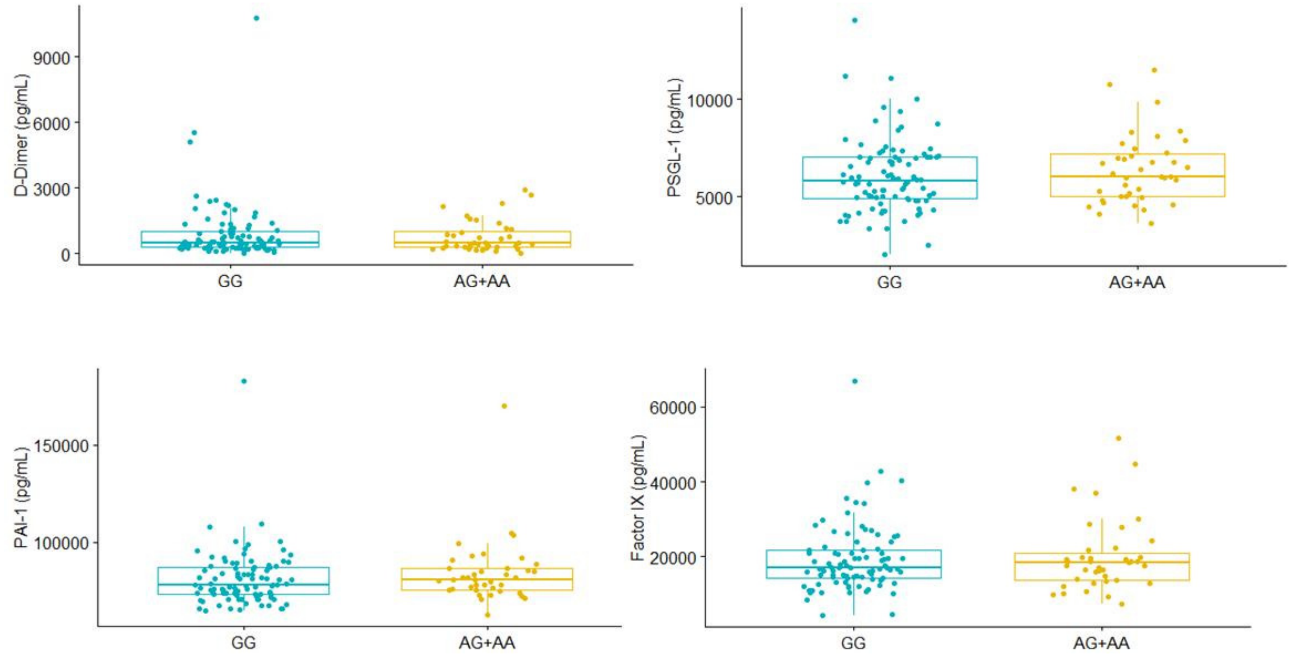

Figure S2. Levels of coagulation proteins (pg/mL) according to *SERPINE1* rs6092 genotype (n=131). Mann Whitney U test, $p>0.05$ in all cases.

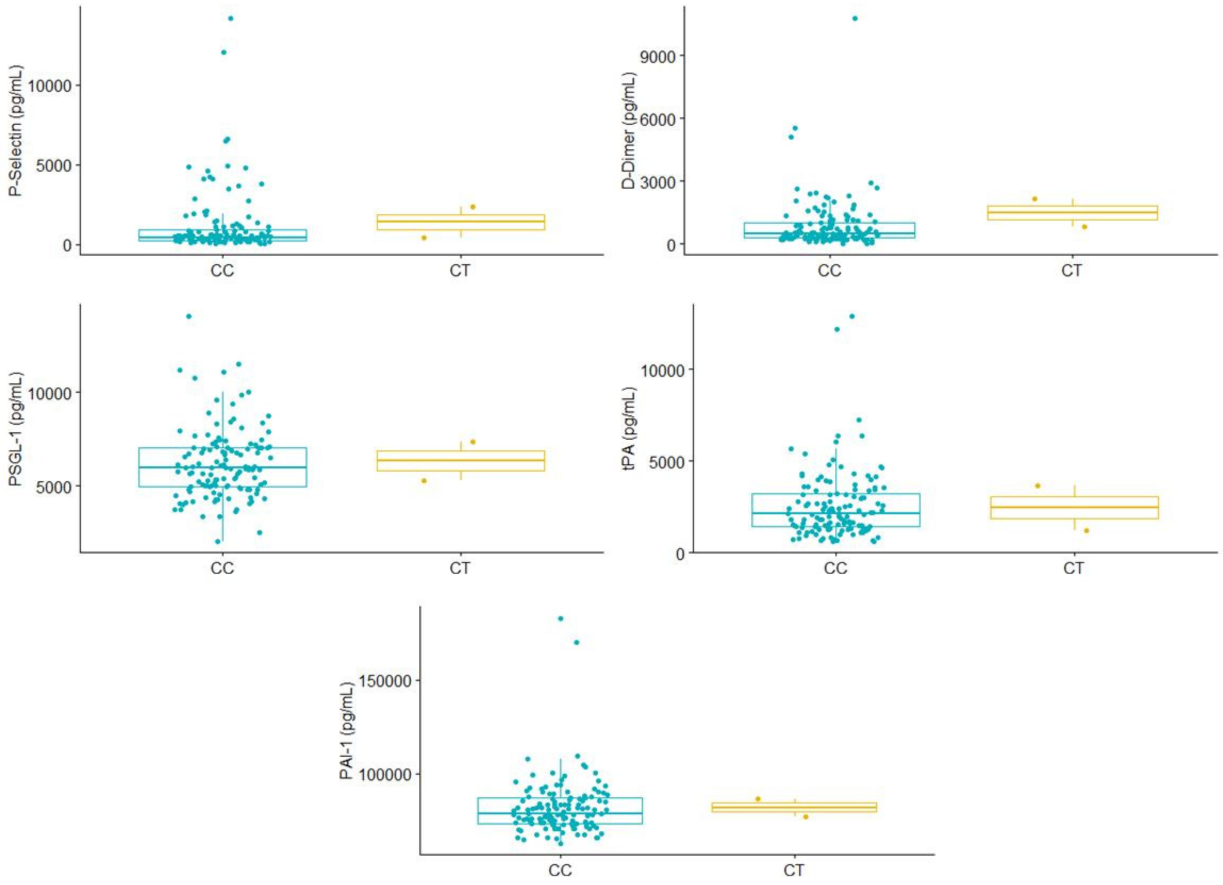

Figure S3. Levels of coagulation proteins (pg/mL) according to *F5* rs6025 genotype (n=131). Mann Whitney U test, *p*>0.05 in all cases.

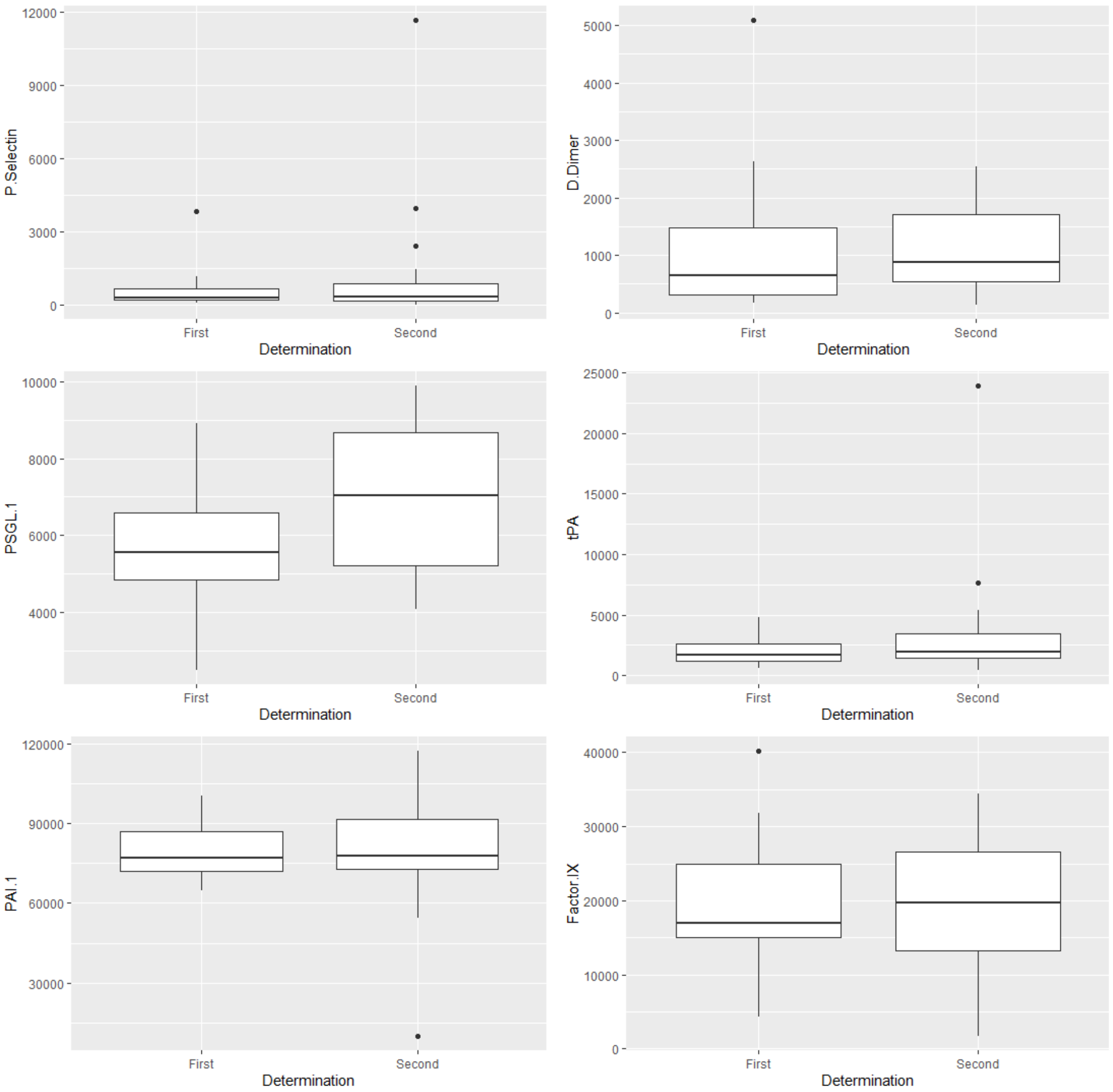

Figure S4. Levels of coagulation proteins (pg/mL) in two determinations for patients with *SERPINE1* rs6092 GG genotype (n=22). Wilcoxon test, $p>0.05$ in all cases.

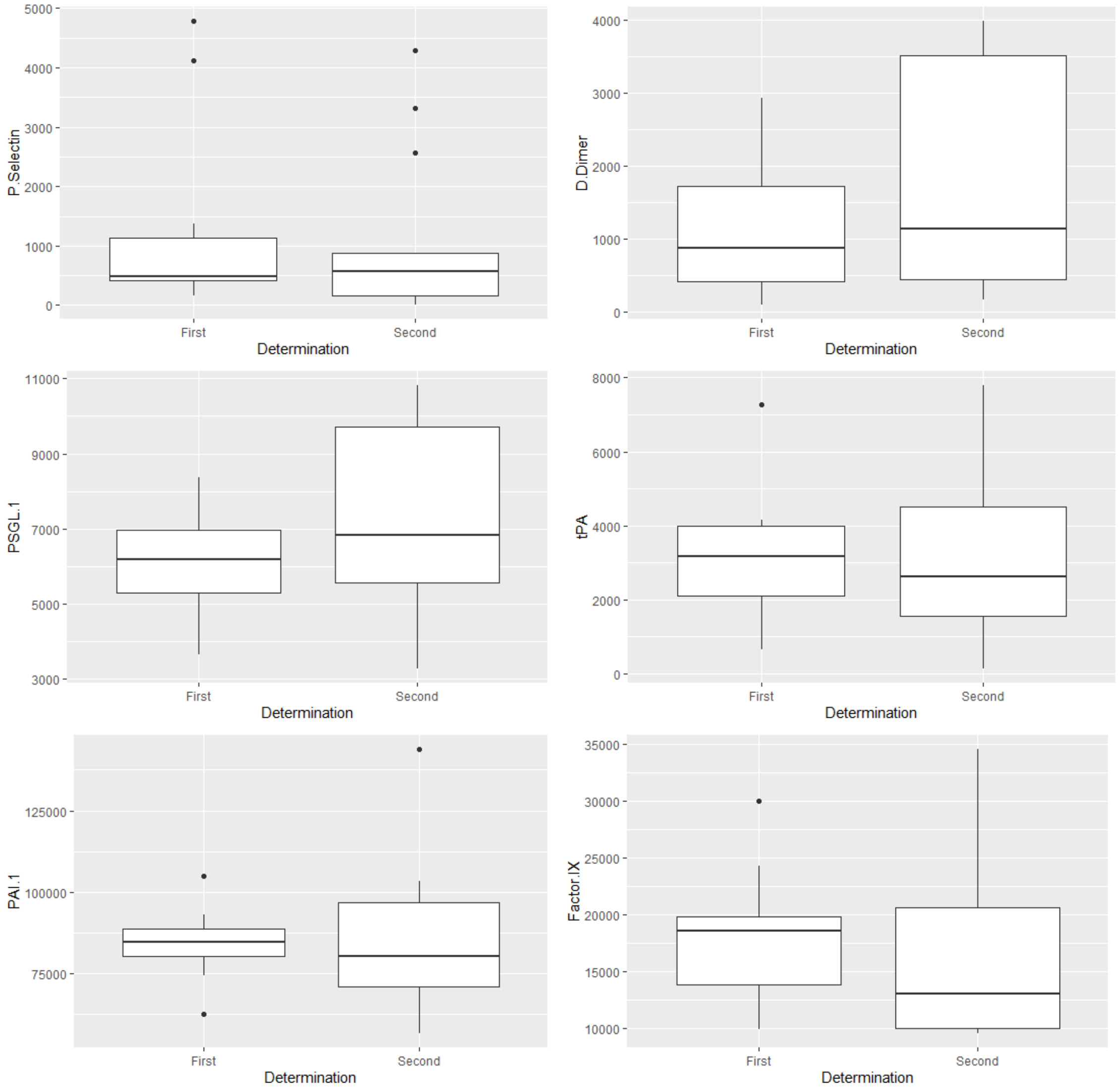

Figure S5. Levels of coagulation proteins (pg/mL) in two determinations for patients with *SERPINE1* rs6092 AG+AA genotype (n=13). Wilcoxon test p>0.05 in all cases.

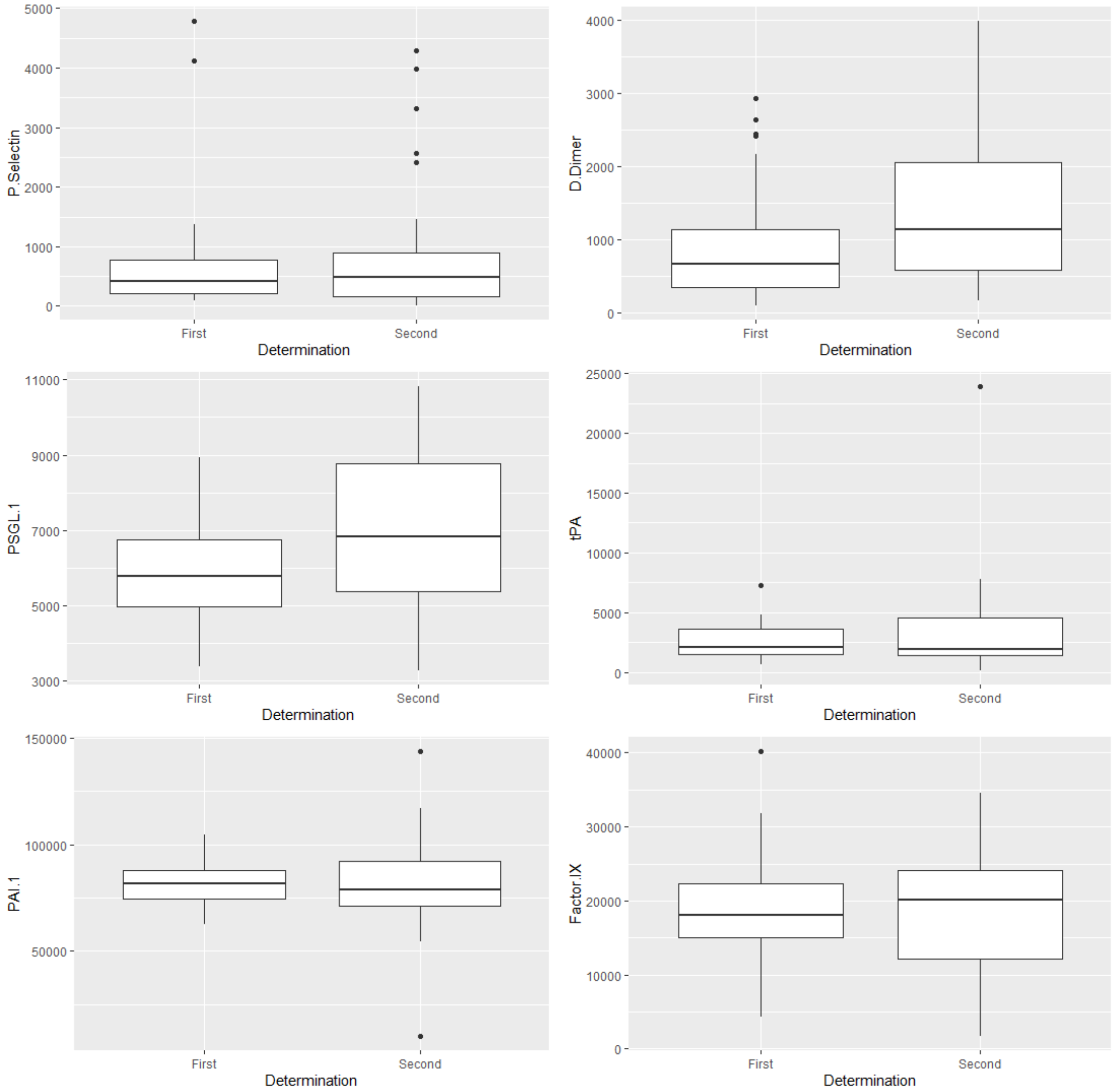

Figure S6. Levels of coagulation proteins (pg/mL) in two determinations for patients requiring invasive mechanical ventilation (IMV, n=29). Wilcoxon test, p>0.05 in all cases.

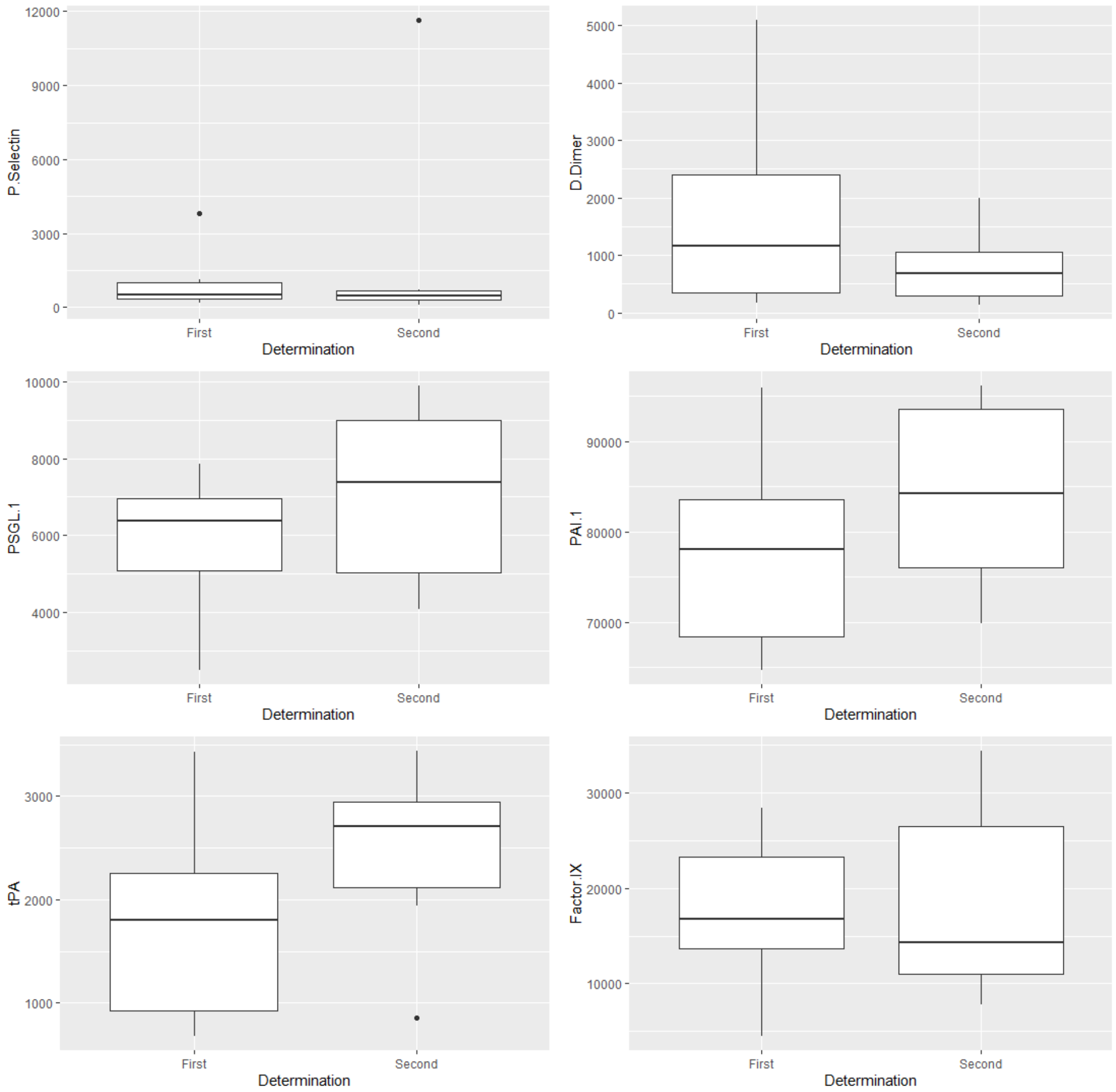

Figure S7. Levels of coagulation proteins (pg/mL) in two determinations for patients who did not require invasive mechanical ventilation (Non-IMV, n=3). Wilcoxon test, p>0.05 in all cases.
